# The 5HTOL/5HIAA Ratio as a Biomarker of Alcohol Hangover

**DOI:** 10.3390/jcm10184241

**Published:** 2021-09-18

**Authors:** Marlou Mackus, Aurora J. A. E. van de Loo, Willie J. M. van den Bogaard, Gerdien A. H. Korte-Bouws, Johan Garssen, Joris C. Verster

**Affiliations:** 1 Division of Pharmacology, Utrecht Institute for Pharmaceutical Sciences, Utrecht University, 3584 CG Utrecht, The Netherlands; marloumackus@gmail.com (M.M.); a.j.a.e.vandeloo@uu.nl (A.J.A.E.v.d.L.); w.j.m.vandenbogaard@uu.nl (W.J.M.v.d.B.); g.a.h.korte@uu.nl (G.A.H.K.-B.); j.garssen@uu.nl (J.G.); 2 Global Centre of Excellence Immunology, Nutricia Danone Research, 3584 CT Utrecht, The Netherlands; 3 Centre for Human Psychopharmacology, Swinburne University, Melbourne, VIC 3122, Australia

**Keywords:** alcohol hangover, biomarker, 5-HIAA, 5-HTOL, serotonin, ethanol

## Abstract

Assessment of the presence and severity of alcohol hangovers relies on the subjective method of self-report. Therefore, there is a need of adequate biomarkers that (1) correlate significantly with hangover severity, and (2) correspond to the level of hangover-related performance impairment objectively. In this naturalistic study, *n* = 35 social drinkers participated. Urine samples were obtained the morning after alcohol consumption and after an alcohol-free control day. Concentrations of 5-hydroxytryptophol (5-HTOL), 5-hydroxyindoleacetic acid (5-HIAA) and the 5-HTOL/5-HIAA ratio were determined. The results confirm previous findings that 5-HTOL and the 5HTOL/5-HIAA ratio are useful biomarkers of recent alcohol consumption. Significant correlations were found with the amount of alcohol consumed, total drink time, and estimated BAC. However, urine concentrations of 5-HTOL and 5-HIAA (and their ratio 5HTOL/5-HIAA) did not significantly correlate with hangover severity. In conclusion, urine 5-HTOL, 5-HIAA, and the 5HTOL/5-HIAA ratio cannot be considered to be suitable biomarkers of alcohol hangover.

## 1. Introduction

The alcohol hangover is the most commonly reported negative consequence of heavy alcohol consumption [1] and refers to the combination of negative mental and physical symptoms which can be experienced after a single episode of alcohol consumption, starting when blood alcohol concentration (BAC) approaches zero [2,3]. Hangovers are characterized by a variety of symptoms including fatigue, headache, and nausea [4], and have a negative effect on cognitive functioning [5]. These effects are reflected in impairments of daily functioning, including driving [6,7,8] and work performance [9]. Regarding the pathology of the alcohol hangover, much remains to be determined [10,11,12], but current evidence suggests that the presence and severity of the hangover is related to the immune response to alcohol consumption [13] and differences in alcohol metabolism [14,15]. Although desired by many consumers, currently there are no hangover treatments marketed for which the effectiveness is scientifically proven [16,17].

Given its functional consequences and impact on potentially dangerous activities such as driving a car, it is desirable that the presence and severity of hangovers can be reliably established. However, currently, self-report is the only way to measure whether one is experiencing an alcohol hangover [18]. This is of concern, as self-report is a subjective assessment that may be influenced by individual viewpoints and experiences, recall bias, and personal perception, which may be affected by circumstances and mood. Previous research into the pathology of the alcohol hangover aimed to discover biomarkers that can objectively demonstrate the presence of alcohol hangover. Biomarkers included, but were not limited to, alcohol metabolites, as well as related substances such as neurotransmitters affected by alcohol consumption, hormones, and cytokines [13,14]. Ideally, the candidate biomarker should also be informative regarding the functional consequences of the hangover state. That is, the concentration of a suitable biomarker should (1) be accurately associated with hangover severity, and (2) correspond to the level of hangover-related performance impairment. These two criteria are, for example, met by breathalyzers assessing breath alcohol concentration in the alcohol intoxication state. The ethanol content in breath or blood correlates highly with (1) reported intoxication and (2) performance of potentially dangerous daily activities such as driving a car [19]. However, it can be questioned if a biomarker for the hangover state can meet both criteria. Whereas ethanol concentrations in urine showed to correlate significantly with hangover severity [20], ethanol can only be detected in a minority of drinkers with self-reported hangover [20,21], and the relationship with performance impairment during the hangover state remains to be determined. In this context, it is important to note that, due to large individual differences, the correlation between self-reported hangover severity and driving impairment was not significant [6]. Thus, the identification of a suitable biomarker for the hangover state that allows the development of a ‘breathalyzer’ for the alcohol hangover state remains an important research goal.

Candidate biomarkers are two metabolites of serotonin, named 5-hydroxytryptophol (5-HTOL) and 5-hydroxyindoleacetic acid (5-HIAA). In particular, the 5-HTOL/5-HIAA ratio is of interest. After consuming alcohol, ethanol is converted rapidly into acetaldehyde, which is the primary metabolite of ethanol. Aldehyde dehydrogenase (ALDH) plays a crucial role in metabolizing acetaldehyde. In this oxidative process in the liver, competitive inhibition of ALDH reduces the binding capacity of serotonin. Furthermore, alcohol consumption increases levels of NADH, favoring the formation of 5-HTOL over 5-HIAA [22]. This shift in serotonin metabolism after alcohol consumption results in an approximate 50-fold increase in the 5-HTOL/5-HIAA ratio [23]. This strong biological response projected by the 5-HTOL/5-HIAA ratio has therefore been previously proposed to serve as biomarker for recent alcohol use [24] and is therefore the subject of the current investigation. Previous research has shown that whereas the 5-HTOL/5-HIAA ratio is usually very low under sober conditions, the ratio in blood and urine is significantly increased after recent alcohol consumption [25] and can be monitored in urine over several hours after ethanol consumption [25]. In fact, both these serotonin metabolites are still present in the urine even after ethanol has been cleared from the body [26,27], which temporally coincides with the onset and duration of hangover symptoms [28,29].

Anecdotal data conservatively indicates that 5-HT3—a receptor binding serotonin—antagonists are useful in alleviating symptoms of alcohol hangovers [30], emphasizing the hypothetical role of serotonin metabolism in the hangover pathology. Bendtsen et al. [23] previously evaluated the potential of the 5-HTOL/5-HIAA ratio to indicate recent drinking during the hangover state. The study described a significant elevation of the 5-HTOL/5-HIAA ratio the day after heavy alcohol consumption and reported a correlation between the 5-HTOL/5-HIAA ratio and severity scores of headache and nausea. Beck and Helander [31] confirmed the functionality of 5-HTOL as a sensitive and reliable marker for recent alcohol intake.

Although these studies showed that the 5-HTOL/5-HIAA ratio is a useful biomarker of recent alcohol consumption, its relevance as biomarker to indicate the presence and severity of the hangover has not been established yet. Therefore, the aim of the current study was to evaluate urinary 5-HTOL, 5-HIAA, and the 5-HTOL/5-HIAA ratio and their possible relationship to the presence and severity of alcohol hangovers.

## 2. Materials and Methods

This naturalistic study comprised a hangover day and a control day (no alcohol consumed the previous day) [32]. Data were collected on test days following real-life drinking sessions. No constraints were imposed upon participants’ behavior in this observational study, and the investigators were not present during the drinking session or on the control day [33]. The University of Groningen Psychology Ethics Committee approved the study (approval code: ppo-013-232, approval date: 7 May 2014), and written informed consent was obtained from all participants.

The *n* = 35 individuals taking part in the study were healthy social drinkers, 18–30 years old, without a history of or current drinking problems. They were recruited via local advertisement. All participants reported having occasions during which they consume at least five alcoholic beverages, with a frequency of at least three times a month. For this study, two types of social drinkers were recruited: individuals who experience hangovers after a night of consuming alcohol (the hangover sensitive group, *n* = 17) and individuals who do not experience hangovers after a night of consuming alcohol (the hangover resistant group, *n* = 18). This distinction was made, since research showed that around 25% of drinkers claim to be hangover resistant [34,35,36,37], i.e., not experiencing a hangover despite consuming the same amount of alcohol as hangover sensitive drinkers. As it can be hypothesized that there are differential effects in the relationship between biomarker assessments and hangover severity, both hangover sensitive and hangover resistant drinkers were included in the current study. The sample size of 18 participants per group was based on a previous research [38]. Assuming 85% of power and a two-sided significance level of 0.05, a sample size of 18 subjects per group would be able to detect a difference in hangover severity scores between the hangover sensitive group and the hangover resistant group of 2, assuming a within-subject standard deviation of 1.5 [32].

Participants consumed alcohol at their own pace and quantity in a setting of their personal choice and preference. In case participants chose not to consume alcohol, the testing days were postponed. Participants were not allowed to consume any form of alcohol at least 24 h before the control day. Additionally, participants were not allowed to consume recreational drugs or caffeinated beverages and foods during the testing days. The absence of drug use (amphetamines, barbiturates, cannabinoids, benzodiazepines, cocaine, and opiates) was verified with InstantView urine drug tests.

Furthermore, participants completed a questionnaire on the number of alcoholic drinks they consumed and the start and stop time of drinking. Their estimated peak blood alcohol concentration (eBAC) on such occasions was computed using the formula of Watson et al. [39], controlling for sex and bodyweight. Overall hangover severity was rated using a 11-point scale, ranging from absent (0) to extreme (10) [18,40]. In addition, using the same scale, the severity of individual hangover symptoms was assessed. These 22 items were derived from the Alcohol Hangover Severity Scale, the Hangover Symptoms Scale, and the Acute Hangover Scale [41,42,43] and included sleepiness, being tired, thirst, headache, concentration problems, nausea, weakness, dizziness, clumsiness, stomach pain, apathy, shaking/shivering, regret, reduced appetite, heart beating, vomiting, confusion, sensitivity to light, heart racing, sweating, anxiety, and depression.

Urine samples of each participant were collected at 09:30 AM on each test day. Urine samples were centrifuged at 3000 rpm for 15 min at room temperature, pipetted into 3 mL cryovials, and stored at a temperature of −20 C. 5-HTOL, 5-HIAA, indole-3-propionic acid and 5-hydroxyindole were purchased from Sigma-Aldrich (St. Louis, MO, USA); acetonitrile and diethyl ether from BioSolve (Valkenswaard, the Netherlands), and other chemicals from Merck (Darmstadt, Germany).

Urine 5-HTOL and 5-HIAA concentrations were determined using high performance liquid chromatography (HPLC) with fluorescence detection. The HPLC system consisted of a Shimadzu LC-10AT pump, an FCV-10AL low pressure gradient valve, a DGU-14A degasser, a SIL-10AD autosampler, a RF-10AXL fluorescence detector and were controlled using Labsolutions software. The separation was achieved using a Synergi Max-RP column, dimensions 150 mm × 4.6 mm × 4 µm (Phenomenex, Torrance, CA, USA) and a mobile phase A consisting of 95 *w*/*w*% 0.1 M sodium acetate buffer pH 5.5 and 5 *w*/*w*% acetonitrile and a mobile phase B consisting of 100% acetonitrile. A gradient elution was used: 0 to 25 min: 95% mobile phase A to 60%A; 25 to 30 min: 100% mobile phase B; 30 to 45min: 95% mobile phase A. The flow rate was set at 0.5 mL/min and the detection was performed at ex/em: 300/350 nm. The urine samples (1.00 mL) were extracted using a modification of the method described by Beck et al. [44]. To the samples was added 200 µl β-glucuronidase (from E. coli K12), 0.1 mL 1 M KH2PO4 pH 6.0 and 100 µl 38 µg/mL indole-3-propionic acid (internal standard, IS). Subsequently, the samples were incubated for 60 min at 37 °C to deglucuronidate the 5-HTOL. After cooling the samples, 0.1 mL 0.25 mg/mL 5-hydroxyindole, 0.1 mL 0.75 M HCl, 1g NaCl and 5.5 mL diethyl ether was added, vortex mixed for 2 min and centrifuged. 4.0 mL of the upper layer was transferred into a clean glass tube and evaporated at 30 Celsius using nitrogen. The samples were reconstituted in 100 µl 15% acetonitrile and 5 µl was injected onto the column. Standard curve samples ranging from 28–7000 ng/mL (5-HIAA) and 1-270 ng/mL (5-HTOL) were prepared in Milli-Q water and extracted as above except no β-glucuronidase was added. Calibration curves were constructed using the peak ratio of 5-HTOL and 5-HIAA to the IS and plotted against their concentration. A weighted regression (1/x) line was calculated and used to calculate the concentrations of the unknown samples. The method was validated according to the US Food and Drug Administration guidelines [45]. Standard solutions were stable for at least six weeks at 4 °C; extracted urine samples were stable for 1 week at room temperature. Linearity of the calibration curves was confirmed and the accuracy (91–104%) and precision (rsd < 10%) were satisfactory. Quality control samples were run in each batch of samples.

Statistical analyses were conducted with SPSS (IBM Corp. Released 2013. IBM SPSS Statistics for Windows, Version 27.0., Armonk, NY, USA). Mean and standard deviation (SD) were computed for all variables. Normality was checked statistically (Kolmogorov–Smirnov test) and by visual inspection. Data were not normally distributed and therefore nonparametric tests were used for the statistical analyses. Comparisons between the post-alcohol day and control day were conducted with the Related Samples Wilcoxon Signed Ranks test. Comparisons between the hangover resistant group and the hangover sensitive group were conducted with the Independent Samples Mann–Whitney U test. Differences were considered significant if *p* < 0.05. Spearman’s rho correlations were computed between alcohol consumption outcomes, hangover severity, and biomarker concentrations. Correlations were considered significant if *p* < 0.05. For individual hangover symptoms, a Bonferroni’s correction was applied to account for multiple correlations, and a *p*-value < 0.0023 (0.05/22) was considered statistically significant.

## 3. Results

Demographics of the participants are summarized in Table 1 and their alcohol consumption outcomes are summarized in Table 2. Individual data of the participants is listed in Appendix A Table A1 and Table A2, respectively. No significant differences between the hangover resistant group and hangover sensitive group were found for demographics or alcohol consumption data.

Participants were allocated to the hangover resistant group at screening, based on historical reporting. However, the reported overall hangover severity in the experiment was 0 (*n* = 12) or 1 (*n* = 6). Therefore, the mean (SD) overall hangover severity score was 0.3 (0.5) and not zero. The reported hangover severity score of the hangover sensitive group was significantly higher (*p* < 0.001) compared to the hangover resistant group.

Outcomes of the biomarker assessments are summarized in Table 3 and Appendix A Table A3.

In the hangover sensitive group, for 5-HIAA no significant differences were found between the control day and the alcohol day. The 5-HTOL concentration (*p* = 0.001) and the 5-HTOL/5-HIAA ratio (*p* = 0.001) were significantly higher on the alcohol day. In the hangover resistant group, for 5-HIAA, no significant differences were found between the control day and the alcohol day. The 5-HTOL concentration (*p* = 0.002) and the 5-HTOL/5-HIAA ratio (*p* < 0.001) were significantly higher on the alcohol day. Comparisons between the hangover resistant group and the hangover sensitive group revealed that the 5-HIAA concentration after the alcohol day were significantly higher in the hangover sensitive group (*p* = 0.022). Other comparisons between the groups were not statistically significant.

Correlations between biomarkers and alcohol consumption outcomes (for the whole sample on the alcohol day) are summarized in Table 4.

Significant correlations were found between the biomarkers and alcohol consumption outcomes. In contrast, overall hangover severity did not correlate significantly with any of the biomarkers. When computing the correlations for the subsample of hangover sensitive participants, the correlations between overall hangover severity and 5-HIAA (r = 0.269, *p* = 0.299), 5-HTOL (r = 0.113, *p* = 0.688), and the 5-HTOL/5-HIAA ratio (r = 0.020, *p* = 0.943) were not statistically significant.

Data for individual hangover symptoms is listed in Appendix A Table A4 and the correlations between biomarkers and the severity of symptoms on the alcohol day is summarized in Table 5.

Except for the correlation between 5-HIAA and headache, none of the correlations between biomarkers and individual hangover symptoms was statistically significant. When conducting the analysis only for hangover sensitive participants, a significant correlation was found between 5-HTOL and thirst (r = 0.686, *p* = 0.002). All other correlations were not statistically significant.

## 4. Discussion

The current findings confirm that 5-HTOL and the 5HTOL/5-HIAA ratio assessed in urine are useful biomarkers of recent alcohol consumption. Our findings are in line with previous studies also suggesting that the 5-HTOL/5-HIAA ratio is a useful biomarker of recent alcohol use [23,24]. In the current study, we found significant correlations with the amount of alcohol consumed, total drink time, and estimated BAC. Further research in a larger population can provide additional support and insights on how to use these metabolites in identifying recent alcohol consumption

Although the 5-HIAA concentration was significantly higher among hangover sensitive drinkers compared to the hangover resistant group, urine concentrations of 5-HTOL and 5-HIAA (and their ratio 5HTOL/5-HIAA) did not significantly correlate with hangover severity. With the exception of correlations between 5-HIAA and headache and between 5-HTOL and thirst, none of the correlations between biomarkers and the severity of individual hangover symptoms were significant. Therefore, it is concluded that urine 5-HTOL, 5-HIAA, and the 5HTOL/5-HIAA ratio cannot be considered to be suitable biomarkers of alcohol hangover.

As with many markers of recent alcohol consumption, these are not found to be reliable indicators of the presence and severity of the hangover. In previous analyses, we found that also ethyl glucuronide (EtG) and ethyl sulfate (EtS), oxidative metabolites of ethanol, were useful to demonstrate recent alcohol use, but their urine concentrations also did not correlate significantly with hangover severity [46]. Whereas acetaldehyde is usually not present in urine during the hangover state, research should continue to evaluate other potential biomarkers of the hangover state. Preferably, these biomarkers should be volatiles, as these can be assessed in breath. In the future, suitable biomarkers can then be used for the development of a breathalyzer for the alcohol hangover state.

Finally, when interpreting the presented data, it is important to take note of some limitations of the study. Firstly, as common to the naturalistic study design, alcohol consumption was not monitored, but self-reported by the participants [33]. This may have resulted in recall bias, affecting the study outcome. Previous night peak BAC was not assessed via breathalyzer, but calculated based on the retrospective reporting of alcohol consumption data. The reported BAC should therefore be regarded as an estimate. The naturalistic study design further implies that both the amount and type of alcoholic beverages consumed differed between participants, as did the activities of participants during their night of drinking (e.g., dancing, talking, drinking at home or in a pub). Even though 5-HTOL and 5-HIAA are not excreted directly after alcohol consumption, voiding during the night may have affected the absolute concentration of these metabolites. The possible consumption of water may have diluted the concentration of 5-HTOL and 5-HIAA, however, this does not affect the computed ratio. Future controlled studies should verify the impact of these behaviors. Secondly, the assessments were made in urine. It should therefore be confirmed by future research whether the association between the biomarkers and hangover severity is also absent in blood. Thirdly, participants were all social drinkers without alcohol dependence. This selection criteria was deemed important as drinkers with alcohol dependence may express different metabolic pathways. It may be interesting to replicate this study in patients with diagnosed alcohol use disorder. Finally, no data was recorded on urine voiding during drinking, at night, and in the morning before the test days. Voiding during the night or in the early morning may have led to the excretion of the biomarkers under investigation. However, as the hangover sensitive and hangover resistant groups both consumed the same amount of alcohol, there is no reason to assume that additional voiding would be different between the two groups. It is however recommended for future studies to record factors that may influence biomarker concentrations, such as additional voiding and water consumption.

Taken together, in the search for a functional biomarker of alcohol hangovers, research has succeeded in excluding several candidates, but has not yet been able to identify a useful and reliable biomarker of the hangover state. In line, the current study showed that 5-HTOL, 5-HIAA, and the 5HTOL/5-HIAA ratio are not suitable as biomarkers of alcohol hangover.

## Figures and Tables

**Table 1:** Demographics and study outcomes.

Demographics	Hangover Resistant Group	Hangover Sensitive Group
N	18	17
Age (year)	20.8 (2.0)	21.4 (1.7)
Height (m)	1.78 (0.1)	1.75 (0.1)
Weight (kg)	71.1 (10.2)	65.8 (10.0)
BMI (kg/m^2^)	22.3 (2.0)	21.4 (2.3)

Mean and standard deviation (SD, between brackets) are shown. Abbreviation: BMI = body mass index.

**Table 2:** Alcohol consumption outcomes on the test day.

Alcohol Outcomes	Hangover Resistant Group	Hangover Sensitive Group
Alcoholic drinks	10.7 (4.7)	11.3 (5.6)
Total drink time (h)	4.9 (2.0)	6.1 (1.8)
Estimated BAC (%)	0.165 (0.07)	0.174 (0.07)
Overall hangover severity	0.3 (0.5)	5.9 (2.0) *

Mean and standard deviation (SD, between brackets) are shown. Significant differences (*p* < 0.05) between the hangover resistant group and hangover sensitive group are indicated by *. Abbreviation: BAC = blood alcohol concentration.

**Table 3:** Biomarker assessments.

Biomarkers	Hangover Resistant Group		Hangover Sensitive Group	
	Control Day	Alcohol Day	Control Day	Alcohol Day
5-HTOL (ng/mL)	17.8 (12.6)	232.9 (343.0) ^γ^	21.3 (15.3)	509.3 (567.8) ^γ^
5-HIAA (µg/mL)	3.8 (2.8)	3.1 (1.4)	4.1 (2.8)	4.6 (2.1) *
5-HTOL/5-HIAA ratio (×1000)	5.6 (2.8)	78.8 (107.3) ^γ^	6.1 (2.8)	126.6 (127.9) ^γ^

Mean and standard deviation (SD, between brackets) are shown. Significant differences (*p* < 0.05) between the hangover resistant group and hangover sensitive group are indicated by *. Significant differences (*p* < 0.05) between the control day and the alcohol day are indicated by ^γ^. Abbreviation: 5-HTOL = 5-hydroxytryptophol, 5-HIAA = 5-hydroxyindoleacetic acid.

**Table 4:** Correlations between biomarkers and alcohol consumption outcomes.

	5-HTOL		5-HIAA		5-HTOL/5-HIAAA Ratio	
Alcohol Outcomes	r	*p*-Value	r	*p*-Value	R	*p*-Value
Number of alcoholic drinks	0.433	0.012 *	−0.199	0.252	0.503	0.003 *
Total drink time (h)	0.411	0.017 *	−0.060	0.733	0.414	0.017 *
Estimated BAC (%)	0.379	0.030 *	−0.161	0.354	0.466	0.006 *
Overall hangover severity	0.229	0.200	0.329	0.054	0.137	0.446

Spearman’s correlations (r) and *p*-values are shown. Significant correlations (*p* < 0.05) are indicated by *. Abbreviations: BAC = blood alcohol concentration, 5-HTOL = 5-hydroxytryptophol, 5-HIAA = 5-hydroxyindoleacetic acid.

**Table 5:** Correlations between biomarkers and the severity of individual hangover symptoms on the alcohol day.

	5-HTOL		5-HIAA		5-HTOL/5-HIAAA Ratio	
Hangover Symptoms	r	*p*-Value	r	*p*-Value	R	*p*-Value
Headache	0.272	0.125	0.524	0.001 *	0.090	0.619
Nausea	0.322	0.068	0.490	0.003	0.188	0.295
Concentration problems	0.297	0.093	0.489	0.003	0.113	0.530
Regret	0.338	0.054	0.473	0.004	0.172	0.337
Sleepiness	0.254	0.154	0.327	0.055	0.172	0.339
Heart beating	0.256	0.150	0.475	0.004	0.111	0.540
Vomiting	0.263	0.139	0.430	0.010	0.113	0.532
Being tired	0.223	0.213	0.326	0.056	0.135	0.455
Shivering/shaking	0.380	0.029	0.202	0.244	0.319	0.070
Clumsiness	0.275	0.121	0.188	0.279	0.219	0.220
Weakness	0.192	0.284	0.282	0.101	0.129	0.475
Dizziness	0.274	0.123	0.321	0.060	0.163	0.364
Apathy	0.235	0.188	0.270	0.117	0.131	0.466
Sweating	0.158	0.379	0.171	0.326	0.101	0.574
Stomach pain	0.144	0.424	0.356	0.036	0.078	0.666
Confusion	0.317	0.073	0.320	0.061	0.216	0.228
Sensitivity to light	0.096	0.594	0.148	0.395	0.032	0.861
Thirst	0.118	0.512	0.304	0.076	0.039	0.830
Heart racing	0.273	0.124	0.242	0.162	0.201	0.262
Anxiety	0.116	0.519	0.235	0.174	0.034	0.853
Depression	0.131	0.468	0.265	0.123	0.047	0.797
Reduced appetite	0.312	0.077	−0.059	0.737	0.360	0.040

Spearman’s correlations (r) and *p*-values are shown. Significant correlations (*p* < 0.0023, after Bonferroni’s correction for multiple comparisons) are indicated by *. Abbreviations: BAC = blood alcohol concentration, 5-HTOL = 5-hydroxytryptophol, 5-HIAA = 5-hydroxyindoleacetic acid.

## Data Availability

The data are listed in Appendix A Table A1, Table A2, Table A3 and Table A4.

## Appendix A

**Table A1:** Demographics—individual participant data.

Participant	Hangover	Age	Height	Weight	BMI
Unit	-	Year	Meter (m)	kg	kg/m^2^
1	resistant	22	1.67	74	26.53
2	resistant	21	1.77	71	22.66
3	resistant	20	1.68	69	24.45
4	resistant	25	1.68	67	23.74
5	resistant	20	1.88	77	21.79
6	resistant	19	1.83	64	19.11
7	resistant	21	1.68	66	23.38
8	resistant	18	1.96	86	22.39
9	resistant	19	1.94	81	21.52
10	resistant	25	1.71	65	22.23
11	resistant	23	1.79	69	21.53
12	resistant	20	1.90	76	21.05
13	resistant	20	1.67	62	22.23
14	resistant	20	1.92	87	23.60
15	resistant	20	1.75	65	21.22
16	resistant	22	1.66	53	19.23
17	resistant	19	1.70	58	20.07
18	resistant	20	1.89	90	25.20
19	sensitive	22	1.70	67	23.18
20	sensitive	21	1.87	83	23.82
21	sensitive	19	1.87	82	23.59
22	sensitive	20	1.69	52	18.21
23	sensitive	20	1.77	60	19.22
24	sensitive	20	1.61	56	21.60
25	sensitive	20	1.68	55	19.49
26	sensitive	24	1.78	75	23.67
27	sensitive	23	1.63	59	22.21
28	sensitive	24	1.76	76	24.54
29	sensitive	20	1.72	73	24.68
30	sensitive	19	1.67	61	21.87
31	sensitive	22	1.68	55	19.49
32	sensitive	21	1.77	57	18.19
33	sensitive	23	1.86	64	18.50
34	sensitive	22	1.80	70	21.60
35	sensitive	23	1.94	72	19.13

Abbreviation: BMI = body mass index.

**Table A2:** Alcohol outcomes—individual participant data.

Participant	Number of Drinks	Drinking Duration	Estimated BAC	Hangover Severity
Unit	Units	Hour	%	0–10
1	5	1.50	0.105	0
2	11	4.75	0.208	0
3	6	3.00	0.114	0
4	8	6.50	0.115	1
5	12	4.50	0.163	0
6	11	6.00	0.146	0
7	17	4.00	0.395	0
8	20	6.00	0.257	0
9	9	3.50	0.115	0
10	7	5.25	0.111	0
11	9	3.00	0.188	0
12	18	9.00	0.208	1
13	5	2.00	0.109	1
14	16	7.25	0.170	1
15	9	3.00	0.183	1
16	6	4.50	0.112	0
17	8	7.00	0.121	1
18	15	7.25	0.148	0
19	12	5.50	0.230	7
20	14	7.50	0.142	6
21	20	9.00	0.233	3
22	10	7.00	0.194	8
23	8	6.00	0.132	7
24	7	4.00	0.142	7
25	8	3.25	0.184	9
26	28	8.33	0.408	8
27	8	5.00	0.151	6
28	12	6.33	0.144	3
29	7	4.00	0.118	7
30	7	5.50	0.113	3
31	7	6.50	0.121	3
32	10	9.00	0.148	4
33	14	8.00	0.250	6
34	10	5.00	0.127	6
35	9	3.67	0.126	8

Abbreviation: BAC = blood alcohol concentration.

**Table A3:** Biomarker assessments—individual participant data.

Participant	5-HTOL		5-HIAA		5-HTOL/5-HIAA Ratio	
	Control Day	Alcohol Day	Control Day	Alcohol Day	Control Day	Alcohol Day
Unit	ng/mL	ng/mL	µg/mL	µg/mL	(ng/mL/µg/mL) × 1000	(ng/mL/µg/mL) × 1000
1	3.56	153.93	2.54	3.42	1.40	44.94
2	16.38	74.89	5.66	5.63	2.89	13.31
3	14.21	13.03	4.32	3.03	3.29	4.30
4	8.06	14.06	0.72	1.78	11.21	7.91
5	17.29	49.00	2.93	3.28	5.91	14.95
6	5.18	633.47	1.04	4.01	5.00	157.97
7	15.26	159.08	2.45	2.06	6.23	77.15
8	13.03	1239.32	3.22	3.72	4.05	333.37
9	34.00	40.82	5.19	4.82	6.55	8.47
10	31.28	97.92	4.73	5.10	6.62	19.19
11	4.01	22.80	0.62	0.65	6.44	35.30
12	7.13	468.57	0.76	3.69	9.43	126.85
13	39.53	16.08	9.22	3.21	4.29	5.02
14	6.76	323.34	1.79	1.33	3.78	243.58
15	12.86	7.12	4.12	0.55	3.12	12.85
16	39.96	56.41	9.77	4.05	4.09	13.92
17	36.44	21.73	7.64	3.07	4.77	7.09
18	15.13	801.49	1.27	2.74	11.89	292.52
19	32.70	557.34	9.14	5.79	3.58	96.25
20	16.78	33.14	7.19	8.57	2.34	3.87
21	27.62	662.81	3.31	2.79	8.36	237.91
22	38.60	1332.52	6.74	4.39	5.72	303.41
23	60.74	1088.98	7.77	4.68	7.85	232.78
24	34.18	59.96	4.47	5.15	7.65	11.63
25	26.25	147.12	5.42	3.28	4.84	44.88
26	9.66	1051.86	4.75	3.33	2.03	316.18
27	ND	259.72	2.73	5.75	ND	45.17
28	3.17	ND	0.41	3.13	7.71	ND
29	15.68	26.00	6.47	7.86	2.42	3.31
30	13.67	1824.73	1.26	5.27	10.85	346.45
31	11.31	109.05	1.51	4.02	7.48	27.12
32	3.46	16.36	0.52	0.48	6.64	33.90
33	21.83	94.45	1.94	4.66	11.26	20.27
34	21.18	376.20	5.27	2.14	4.02	176.07
35	4.20	ND	0.76	7.36	5.52	ND

ND = not determined. Abbreviations: 5-HTOL = 5-hydroxytryptophol, 5-HIAA = 5-hydroxyindoleacetic acid.

**Table A4:** Individual hangover symptoms, assessed on the alcohol day—individual participant data.

Participant	Headache	Nausea	Concentration Problems	Regret	Sleepiness	Heart Beating
1	0	0	1	0	2	0
2	2	0	4	0	6	0
3	0	0	0	0	0	0
4	0	0	1	0	3	0
5	0	0	0	0	0	0
6	0	0	0	0	2	0
7	0	0	3	0	2	0
8	0	1	0	0	2	0
9	0	0	0	0	0	0
10	0	0	0	0	1	0
11	0	0	1	0	2	0
12	2	0	0	0	0	0
13	0	0	2	0	1	0
14	0	0	0	0	2	0
15	0	0	0	0	4	0
16	0	0	2	0	2	0
17	2	1	2	0	4	0
18	0	0	3	1	4	0
19	4	5	9	7	8	8
20	5	6	5	2	5	4
21	3	0	4	0	5	0
22	8	9	6	8	10	3
23	7	8	7	1	7	0
24	9	6	5	1	6	4
25	10	9	8	5	8	4
26	8	8	5	3	9	4
27	6	6	7	5	9	0
28	2	0	3	0	4	0
29	8	6	7	2	7	2
30	2	3	3	0	2	3
31	3	1	4	1	3	0
32	2	2	2	0	5	0
33	5	2	3	0	7	0
34	1	2	3	1	4	1
35	9	8	7	8	8	5
**Participant**	**Vomiting**	**Being Tired**	**Shaking/** **Shivering**	**Clumsiness**	**Weakness**	**Dizziness**
1	0	2	0	2	0	0
2	0	6	0	2	0	0
3	0	1	0	0	0	0
4	0	2	0	1	1	0
5	0	1	0	0	0	0
6	0	3	0	0	0	0
7	0	3	3	3	2	3
8	0	3	0	0	0	0
9	0	0	0	0	0	0
10	0	2	0	0	0	0
11	0	2	0	0	0	0
12	0	2	0	0	0	0
13	0	1	0	0	0	0
14	0	2	0	1	1	0
15	0	6	0	1	1	0
16	0	2	0	0	0	0
17	0	4	0	2	2	0
18	0	3	0	0	4	0
19	6	8	10	7	8	6
20	4	6	1	2	6	2
21	0	5	0	1	0	0
22	8	9	7	8	5	7
23	1	8	2	5	5	4
24	0	7	0	3	6	6
25	8	9	2	7	8	8
26	5	7	4	6	6	8
27	0	8	0	6	8	2
28	0	2	0	0	0	0
29	2	9	1	1	3	3
30	0	1	4	1	1	0
31	0	2	1	1	1	2
32	0	7	1	2	4	1
33	0	7	6	0	7	0
34	0	3	4	5	3	3
35	2	9	0	2	6	5
**Participant**	**Apathy**	**Sweating**	**Stomach Pain**	**Confusion**	**Sensitivity to Light**	**Thirst**
1	2	0	0	0	0	4
2	2	0	0	0	0	1
3	0	0	0	0	0	0
4	0	0	0	0	1	2
5	0	0	0	0	0	1
6	0	0	0	0	0	0
7	0	0	0	1	0	3
8	0	0	0	1	0	2
9	0	0	0	0	0	0
10	0	0	0	0	0	1
11	0	0	0	0	0	3
12	0	0	0	0	0	4
13	0	0	0	0	0	4
14	0	0	0	0	0	3
15	0	0	0	0	0	7
16	0	0	0	0	0	2
17	1	0	0	0	0	2
18	5	2	0	0	6	6
19	3	4	7	4	4	8
20	4	1	6	4	4	8
21	0	0	0	0	0	3
22	7	3	3	2	0	6
23	4	0	0	0	3	6
24	2	0	7	0	3	8
25	7	1	9	6	7	8
26	7	2	7	8	3	2
27	0	0	0	1	0	8
28	2	0	0	0	0	3
29	1	6	1	1	1	7
30	0	0	1	0	0	3
31	1	0	0	1	0	5
32	3	1	4	0	1	3
33	1	0	2	0	0	6
34	1	0	1	3	3	4
35	8	0	7	3	2	10
**Participant**	**Heart Racing**		**Anxiety**	**Depression**	**Reduced Appetite**	
1	0		0	1	0	
2	0		0	0	0	
3	0		0	0	0	
4	0		0	0	0	
5	0		0	0	0	
6	0		0	0	0	
7	0		0	0	0	
8	0		0	0	0	
9	0		0	0	0	
10	0		0	0	0	
11	0		0	0	0	
12	0		0	0	0	
13	0		0	0	0	
14	0		0	0	0	
15	0		0	0	0	
16	0		0	0	0	
17	0		0	0	2	
18	0		0	0	2	
19	8		3	3	2	
20	5		3	3	0	
21	0		0	0	0	
22	0		0	0	4	
23	0		0	0	4	
24	0		0	0	0	
25	2		3	0	8	
26	4		2	2	6	
27	0		0	0	0	
28	0		0	0	4	
29	0		0	0	0	
30	4		0	0	0	
31	1		0	0	0	
32	0		0	0	4	
33	0		0	0	0	
34	1		0	0	2	
35	0		0	0	9	

Scores range from 0 (absent) to 10 (extreme).
